# New Books

**Published:** 2004-08

**Academic Health Centers: Leading Change in the 21st Century**

Linda T. Kohn, ed.

Washington, DC:National Academies Press, 2004. 216 pp. ISBN: 0-309-08893-3, $39

**Bioethics in Complexity: Foundations and Evolutions**

Sergio De Risio, Franco F. Orsucci, eds.

River Edge, NJ:World Scientific, 2004. 100 pp. ISBN:1-86094-399-3, $34

**Biological Weapons Defense: Infectious Disease and Counterbioterrorism**

Luther E. Lindler, Frank J. Lebeda, George Korch

Totowa, NJ:Humana Press, 2004. 625 pp. ISBN: 1-58829-184-7, $145

**Bioterrorism and Food Safety**

Barbara A. Rasco, Gleyn E. Bledsoe

Boca Raton, FL:CRC Press LLC, 2004. 296 pp. ISBN: 0-8493-2787-3, $99.95

**Cell Growth: Control of Cell Size**

Michael N. Hall, Martin Raff, George Thomas, eds.

Woodbury, NY:Cold Spring Harbor Press, 2004. 652 pp. ISBN: 0-87969-672-9, $135

**Chemical Consequences: Environmental Mutagens, Scientist Activism, and the Rise of Genetic Toxicology**

Scott Frickel

Piscataway, NJ:Rutgers University Press, 2004. 224 pp. ISBN: 0-8135-3412-7, $22.95

**Earthly Politics: Local and Global in Environmental Governance**

Sheila Jasanoff , Marybeth Long Martello, eds.

Cambridge, MA:MIT Press, 2004. 376 pp. ISBN: 0-262-10103-3, $67

**Environmental Contaminants**

Daniel Vallero

Burlington, MA:Elsevier, 2004. 832 pp. ISBN: 0-12-710057-1, $79.95

**Folding and Self-Assembly of Biological Macromolecules**

E. Westhof, N. Hardy

River Edge, NJ:World Scientific, 2004. 416 pp. ISBN: 981-238-500-2, $142

**Free Radicals and Inhalation Pathology**

Erich Schiller

New York:Springer-Verlag, 2004. 773 pp. ISBN: 3-540-00201-4, $225

**Green Giants? Environmental Policies of the United States and the European Union**

Norman J. Vig, Michael G. Faure, eds.

Cambridge, MA:MIT Press, 2004. 392 pp. ISBN: 0-262-22068-7, $67

**Molecular Basis of Breast Cancer: Prevention and Treatment**

Jose Russo, Irma H. Russo

New York:Springer-Verlag, 2004. 448 pp. ISBN: 3-540-00391-6, $249

**Nature and Human Communities**

T. Sasaki, ed.

New York:Springer-Verlag, 2004. 216 pp. ISBN: 4-431-20720-1, $99

**Reviews of Environmental Contamination and Toxicology**

George Ware, ed.

New York:Springer-Verlag, 2004. 202 pp. ISBN: 0-387-20519-5, $129

**The Cancer Handbook**

Malcolm R. Alison, ed.

Hoboken, NJ:John Wiley & Sons, 2004. 1,772 pp. ISBN: 0-470-02506-9, $495

**The Economics of Groundwater Remediation and Protection**

Paul E Hardisty, Ece Ozdemiroglu, Jonathan Smith

Boca Raton, FL:CRC Press LLC, 2004. 400 pp. ISBN: 1566706432, $149.95

**The Practical Bioinformatician**

Limsoon Wong, ed.

River Edge, NJ:World Scientific, 2004. 540 pp. ISBN: 981-238-846-X, $100

**Urban Transport and the Environment**

Edited by World Conference on Transport Research Society and the Institute for Transport Policy Studies

Burlington, MA:Elsevier, 2004. 450 pp. ISBN: 0-08-044512-8, $88

**Water and Sustainable Development: Opportunities for the Chemical Sciences–A Workshop Report to the Chemical Sciences Roundtable**

Parry Norling, Frankie Wood-Black, Tina M. Masciangioli, eds.

Washington, DC:National Academies Press, 2004. 106 pp. ISBN: 0-309-09200-0, $22.95

